# A Hypothesis Regarding Neurosecretory Inhibition of Stress Mediators by Colchicine in Preventing Stress-Induced Familial Mediterranean Fever Attacks

**DOI:** 10.3389/fimmu.2022.834769

**Published:** 2022-02-17

**Authors:** Cengiz Korkmaz, Döndü Üsküdar Cansu, Güven Barıs Cansu

**Affiliations:** ^1^ Department of Internal Medicine, Division of Rheumatology, School of Medicine, Eskisehir Osmangazi University, Eskisehir, Turkey; ^2^ Department of Endocrinology, School of Medicine, Kutahya Health Science University, Kutahya, Turkey

**Keywords:** familial Mediterranean fever, stress, catecholamines, microtubule system, axonal transport, norepinephrine, epinephrine

## Abstract

Familial Mediterranean fever (FMF) is a monogenic autoinflammatory disease characterized by recurrent episodes of fever and serositis. Colchicine (Col) has a crucial role in the prevention of amyloidosis and FMF attacks. The effect of Col on innate immune cells is based on the inhibition of the microtubule system. The microtubule system is also very important for neurosecretory functions. The inhibitory effect of Col on neurosecretory functions is an overlooked issue. Considering that the neuroimmune cross-talk process plays a role in the development of inflammatory diseases, the effect of Col on the neuronal system becomes important. FMF attacks are related to emotional stress. Therefore, the effect of Col on stress mediators is taken into consideration. In this hypothetical review, we discuss the possible effects of Col on the central nervous systems (CNS) and peripheral nervous systems (PNS) in light of mostly experimental study findings using animal models. Studies to be carried out on this subject will shed light on the pathogenesis of FMF attacks and the other possible mechanisms of action of Col apart from the anti-inflammatory features.

## Introduction

Familial Mediterranean fever (FMF) is a common, Mendelian-inherited monogenic autoinflammatory disease characterized by irregular attacks of paroxysmal fever and serositis (1). AA amyloidosis is a dreadful complication of FMF. Colchicine (Col) has been shown to prevent attacks and the development of amyloidosis (2), but when given during FMF attacks, Col cannot end the attacks (2, 3). We do not know the exact reason for this situation. However, it is interesting to note that Col has been shown to alleviate gout arthritis attacks within a short time (3). It is well known that gout, similar to FMF, is a disease that develops due to inflammasome activation and benefits from interleukin-1 (IL-1) receptor antagonist drugs (4). Why does Col not act immediately in FMF attacks as it does in gout attacks? Some researchers have tried to explain why Col does not act immediately in FMF attacks. Ben Chetrit et al. suggested that the immediate anti-inflammatory effect of Col requires interaction with tubulins, which occurs at a low dose of Col. However, the inhibition of genes involved in inflammation at the transcriptional level occurs at higher doses and is observed after 12-24 hours. They suggested that these reasons might explain why Col is not effective when given during the occurrence of an FMF attack (5). However, we do not know exactly whether these inflammatory genes, apart from caspase-1 (CASP-1), have a specific role in the development of FMF attacks. Furthermore, in this study, the authors used human umbilical vein endothelial cells (HUVECs) to study the effects of Col on global gene expression. Although neutrophils and HUVECs may have similar repertoires of expressed genes, their behaviour towards an inflammatory stimulus is different. For instance, exposure to epinephrine (EPI) *in vitro* increases IL-8 expression and CD11b (alpha M integrin) levels in human neutrophils (6). On the other hand, EPI facilitates the downmodulation of adhesion molecule expression in HUVECs, reducing neutrophil adhesion (7). Moreover, in contrast to the abovementioned study, Col increased proinflammatory gene expression *in vitro* in neutrophils and showed its anti-inflammatory effect only through the inhibition of CASP-1 activation (8). These observations suggest the requirement of another explanation of why Col is ineffective when given during an FMF attack.

We believe that it would be more appropriate to look at this mystery in the context of triggers that initiate FMF attacks, and the effect of Col should be evaluated within this context. Approximately two-thirds of patients with FMF reported that their attacks were triggered by emotional stress or other psychological causes (9–17). Interestingly, years ago, the triggering of FMF attacks with the synthetic sympathomimetic drug metaraminol, which was used as a diagnostic test (18), suggested that there might be a relationship between inborn error of catecholamine metabolism and FMF attacks. In one study, selective serotonin reuptake inhibitors reduced the attack frequency in patients with colchicine-unresponsive FMF who also had depression (13). The European Alliance of Associations for Rheumatology (EULAR) stated that periods of physical or emotional stress can trigger FMF attacks, and it may be appropriate to increase the dose of Col temporarily (19). The most important question to be asked here is that if emotional stress is a factor in the emergence of FMF attacks, is the prophylactic effect of Col due to its effect on the sympathoadrenal system in addition to its anti-inflammatory effects?

The role of Col at this stage is its connection with the microtubule system. Microtubules are key parts of cellular functions, including the maintenance of cell shape, cell migration, and cytokine release and transport (20). Microtubules are indispensable in the axonal transport of catecholamines and other amines in the central nervous system (CNS) and peripheral nervous systems (PNS) (21). The effects of Col have been explained by its action on tubulin disruption, which causes the downregulation of multiple inflammatory pathways and the modulation of innate immunity (22). This review outlines the role of Col in the CNS and PNS in terms of its neurosecretory inhibitor role and discusses the prophylactic effect of Col on stress-induced FMF attacks through this mechanism, in addition to its anti-inflammatory properties.

## Stress as a Stimulator of Neuroimmune Cross-Talk Process

Stress, in an ‘integrated definition’, is ‘a group of events, consisting of a stimulus (stressor), that triggers a reaction in the brain (stress perception), that activates the physiologic fight or flight system in the body’ (23). Following exposure to emotional stress, the sympathetic nervous system (SNS) is activated and releases EPI and norepinephrine (NE) into the circulation. These stress-responsive hormones affect the cardiovascular, musculoskeletal, neuroendocrine and immune systems to elicit ‘fight or flight’ action (23). It is well known that there is neural-immune cross-talk in inflammatory diseases. Neuroanatomic and neurochemical studies have shown that primary and secondary lymphoid organs such as the bone marrow, thymus, spleen, lymph nodes, and gut are innervated by sympathetic nerve fibres (24). The activation of postganglionic splenic sympathetic nerve fibres leads to the release of NE at the neuroimmune junction (25). The stress hormones EPI and NE released from both the CNS and PNS can activate inflammatory pathways, including the pyrin inflammasome, by binding to G protein-coupled receptors (GPCRs) on inflammatory cells. GPCRs belong to the largest cell surface receptor family and regulate intracellular signalling cascades in response to neurotransmitters and hormones (26). Ras homologous protein guanosine triphosphate (RhoA) modification is one of the most important roles of GPCRs in FMF (27). As mentioned below, RhoA alteration can activate or inhibit the pyrin inflammasome (28).

What evidence exists that stress causes inflammation in healthy people? Laboratory studies showed that acute stress was associated with significant increases in IL-1β, IL-1 receptor antagonist, IL-6, and TNF-α (29). Bierhaus et al. looked into the mechanisms that turn psychosocial stress into mononuclear cell activation (30). NF-kB was immediately elicited by stress exposure in 17 of 19 individuals, along with higher levels of catecholamines and cortisol, and recovered to baseline within 60 minutes. In two patients who did not have an increase in catecholamines and cortisol, there was no increase in NF-kB binding activity. Another study found that acute psychologic stress elevated circulating levels of IL-6 and IL-1β, and that β blockers could attenuate this reaction (31).

However, we can’t say for sure that stress mediators EPI and NE produce inflammation in all tissues and under all circumstances. These effects are influenced by both tissue and environmental factors. In an experimental arthritic animal model, EPI can lower the expression of TNF-alpha, IL-1β, and IL-6 (32). However, alpha adrenergic receptors are important in neutrophil migration and peritoneal tissue inflammation caused by monosodium urate crystals (33). These examples demonstrate that catecholamines can have a variety of tissue- and condition-specific effects.

Various studies have looked into the impact of the immune system on the neural system, which is the opposite side of neuroimmune cross-talk. Immune system activation is accompanied by alterations in hypothalamic, autonomic, and endocrine systems. IL-1 has been identified as a key cytokine that transports immune activation to the brain in several studies (34, 35). Il-1 has been shown to influence hypothalamic neurosecretory activity and enhance the turnover of NE in the hypothalamus (36–38). Il-1 also has an effect on the hypothalamus’s synthesis of corticotropin-releasing hormone (CRH). CRH can then influence the HPA axis, causing stress hormone levels to rise (39, 40). In the CNS, IL-1 can activate a number of downstream pathways, including the MAPK and NF-kB pathways (41, 42). IL-1, IL-2, IL-6, IFN-γ, and TNF-α all regulate HPA axis activation, which is influenced by glucocorticoid release (43).

## The Effect of Colchicine on the Central Nervous System

It is well known that the SNS and the hypothalamic-pituitary-adrenocortical (HPA) axis are two major pathways that mediate a close relationship between the CNS and immune system (IS) (24). Both pathways are controlled by limbic and autonomic tracks known to mediate the effects of stressors on body functions (24). Sympathetic activation either pharmacologically or through physical or psychosocial stressors can significantly alter immune function (44). Stimulating or blocking the activity of autonomic/limbic brain regions/nuclei known to regulate autonomic/neuroendocrine outflow also alters measures of immune function (45). The inhibitory effects of Col on axonal flow and various types of secretion are well known. In light of this information, it would be reasonable to examine whether the effect of Col may play a preventive role in stress-related FMF attacks.

Although Col has a lipophilic structure, its brain-blood barrier (BBB) penetration is very low (46). A pump protein located on the luminal side of the BBB called P-170 glycoprotein (P-GP) limits the transport of Col across the BBB (47). In a post-mortem study of patients who died due to Col toxicity, Col was detected at 5 ng/g in brain tissue, 396 ng/g in the kidney, and 347 ng/g in the liver (48). On the other hand, a toxicokinetic study reported that the Col level was greater than 600 ng g-1 in bone marrow, 400 ng g-1 in the testicles, 250 ng g-1 in the spleen, and 200 ng g-1 in the kidneys. Col was also found in the brain at 125 ng g-1 (49). As a result, the transport of Col to brain tissue is less than that to other tissues. However, despite this low transportation, Col may be useful in some intracranial inflammatory conditions. For example, Col may have a beneficial effect in patients with FMF with recurrent meningitis called Mollaret’s meningitis (50, 51). This example demonstrates that Col, even at therapeutic doses, may be beneficial in FMF-related intracerebral inflammatory conditions.

Neurosecretory functions in the brain occur through axonal transport, and brain functions are dependent on this process. The microtubule system plays an important role in these neurosecretory functions. It is known that the paraventricular nuclei (PVN) receive dense catecholaminergic innervation originating from noradrenergic and adrenergic cell groups in the lower brain stem (52, 53). The functional effects of Col on the entire neurosecretory tract and various hypothalamic nuclei have been studied. One of the actions of Col is the inhibition of the proximo-distal transport of catecholamine-containing dense-core vesicles. The perturbation in axonal flow was shown by the accumulation of neurosecretory material (53). It is well known that reserpine is an adrenergic blocking agent used to treat mild-to-moderate hypertension *via* the disruption of NE vesicular storage. The antihypertensive action of reserpine is a result of its ability to deplete catecholamines from peripheral sympathetic nerve endings. Similar to reserpine, Col also inhibits central catecholamine release. However, this occurs later with Col than with reserpine (54). There are experimental studies in the literature that strengthen this view (55–57). Abdelmalek et al. reported a decrease in blood pressure by injecting Col into the “anterior forebrain” of rats. They argued that this developed through an inhibition of the central component of the SNS by Col (55). Col achieves these effects by disrupting the transport of NE in central noradrenergic neurons (56). Intrahypothalamic administration of Col may cause behavioural changes in rats. The reason for this is the disruption of catecholamine transport by Col (57). In conscious rats, the administration of Col into the diagonal band of Broca, which has been shown to play an important role in the regulation of hydromineral balance, and in particular, in the regulation of arterial pressure, decreases the arterial pressure responses to both alpha-2 (α-2) and β-adrenergic agonists. The authors have explained this situation by a deterioration of the neural information process by Col (58). In conclusion, Col may limit catecholamine discharge by disrupting the neurosecretory transport process through its effect on the microtubule system in the CNS.

## The Effect of Colchicine on the Peripheral Nervous System and Adrenal Glands

Under stress, NE is released from the sympathetic ganglia, while EPI and dihydroxyphenylalanine (DOPA) are secreted from the adrenal medulla. Col prevents the release of neurotransmitters, particularly noradrenaline, from stimulated sympathetic fibres (59) and similarly inhibits catecholamine release from the adrenal medulla (60). It has been reported that electrical stimulation of the hypogastric nerve increases the release of both dopamine beta-hydroxylase and NE (59). However, when the test was repeated after Col was given to the experimental animals, the release of both mediators was completely inhibited. Such an inhibitory effect of Col has been reported in various types of secretions: insulin (61), histamine from mast cells (62), and collagen from osteoblasts (63, 64). As a result, these kinds of studies at the molecular level have led to the use of Col in various diseases. For instance, Col can be used as an antifibrotic or antiurticarial drug due to these kinds of effects in clinical practice (65, 66).

Balanced function of the sympathetic and parasympathetic nervous systems is very important in cardiac electrophysiology. Cardiac autonomic functions can be evaluated with the heart rate recovery index (HRRI), heart rate variability (HRV), heart rate turbulence (HRT), and QT dynamics (67). Tochinai et al. showed that the RR and QRS intervals were prolonged on electrocardiography (ECG) in rats exposed to Col. They also found an increase in the high-frequency component as evidence of an increase in parasympathetic tone (68). No studies have directly evaluated the effect of Col on the autonomic nervous system in humans. However, some studies have evaluated cardiac autonomic status in patients with FMF using Col. Rosenbaum et al. showed that some of the patients with FMF had autonomic dysfunction in the form of postural tachycardia syndrome and cardioinhibitory and orthostatic hypotension due to dysautonomia independent of amyloidosis. The authors did not comment on whether Col played a role in these results (69). In another study, delayed recovery of heart rate and abnormal HRV and HRT parameters were found in attack-free patients with FMF. Although the authors attributed these changes to the inflammatory feature of the disease, they did not discuss whether Col would affect these results (67). However, contrary to these studies, some studies did not detect a problem related to dysautonomia in FMF patients (70, 71). The results in the clinical studies are inconsistent with each other because of the heterogeneity of demographic characteristics, differences in Col dose, and other disease characteristics of FMF patients. Therefore, studies that directly evaluate the effect of Col on cardiac autonomic functions by eliminating all other confounding factors are needed.

On the other hand, examples found in Col-related toxicity reports can provide some clues. For example, patients with Col toxicity may experience hypotension due to a decrease in vascular resistance. One of the cardiac effects of Col is bradycardia, and this side effect has been explained by the predominance of the parasympathetic system (72, 73). However, arrhythmias and tachycardia were also detected in some patients.

Barakat et al. were the first researchers to mention the role of catecholamines in the pathogenesis of FMF and to show that Col changes catecholamine levels in FMF patients. They found lower plasma EPI and NE levels in FMF patients using Col than in those not using Col (74). Moreover, urinary metanephrine levels were also lower in patients using Col. Again, the same investigators found that dopamine beta-hydroxylase (DBH) activity was higher in untreated attack-free patients and patients with attacks than in healthy controls. They have also shown that Col reduced DBH activity (75). These results suggest that the sympathoadrenal system may have a role in the pathogenesis of FMF and that Col interferes with this process.

## The Anti-Inflammatory Effects of Colchicine on FMF-Related Inflammation

**Figure 1:**
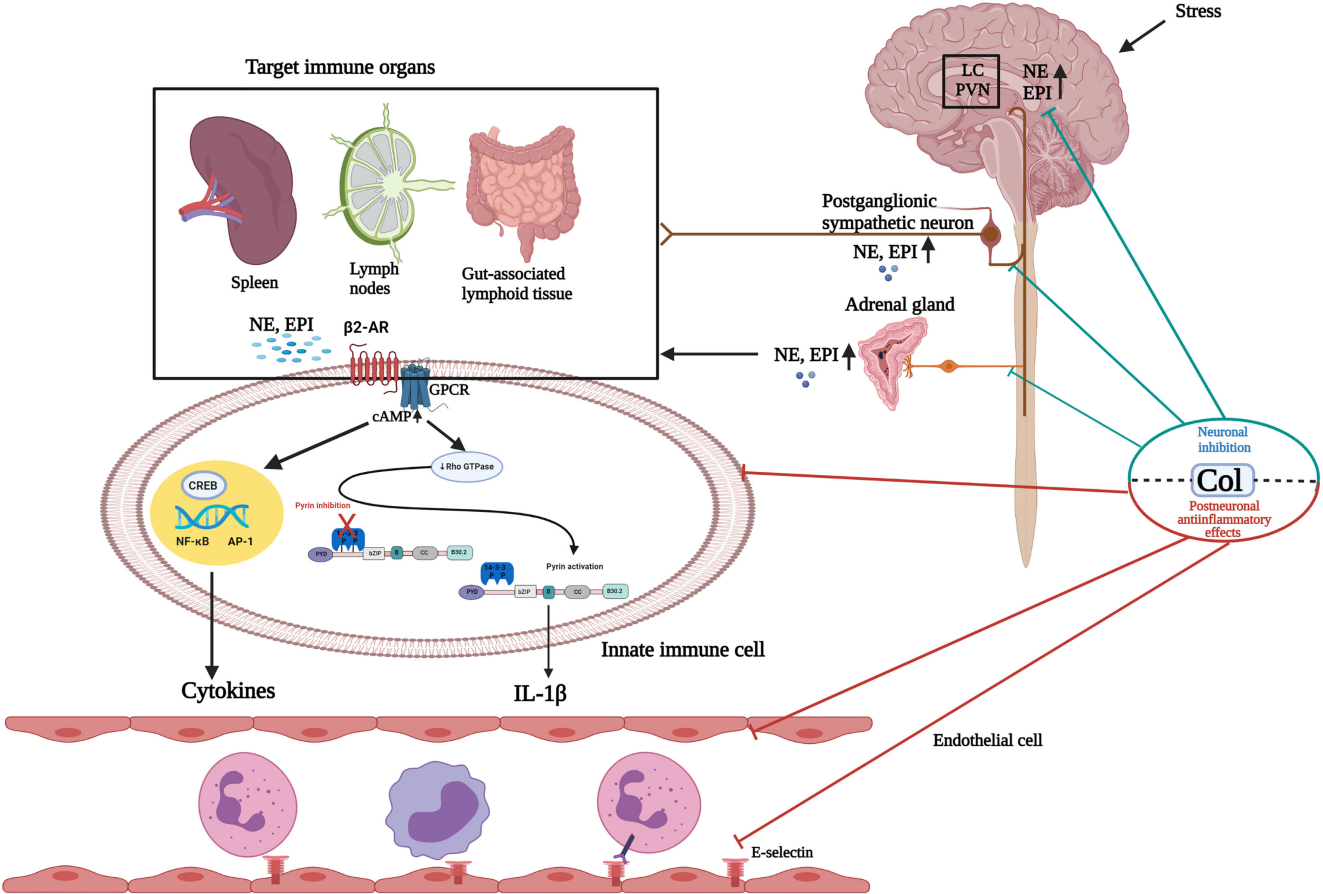
Schematic illustration of the effects of colchicine on the sympathoadrenal medullar axis and postneuronal inflammatory pathways. Paraventricular nuclei (PVN) and the locus coeruleus (LC) receive dense catecholaminergic innervation originating from noradrenergic and adrenergic cell groups in the lower brain stem. Following exposure to stress, the hypothalamic-pituitary adrenal (HPA) axis and sympathoadrenal medullar axis (SAM) are activated. In the SAM axis, preganglionic sympathetic neurons secrete acetylcholine in the adrenal medulla, which stimulates the release of catecholamines, predominantly epinephrine (EPI) and, to a much lesser extent, norepinephrine (NE), into the circulation. Postganglionic sympathetic neurons also predominantly secrete NE. It is well known that sympathetic noradrenergic nerves innervate primary and secondary lymphoid organs. Circulating NE/EPI can potentiate the actions of sympathetic nerves acting at adrenergic receptors in immune organs. These stress mediators may activate the inflammasome and other inflammation pathways by binding to adrenergic receptors expressed on immune cells in a synapse-like fashion or following diffusion through the parenchyma of the innervated tissue. Colchicine (Col), at the neuronal level, inhibits catecholamine transportation through the CNS and PNS and prevents the release of EPI from the adrenal glands (neuronal effect). At the postneuronal level, Col keeps the pyrin inflammasome in an inactive form by activating RhoA. Thus, it inhibits caspase-1 activation and IL-1β release. Col inhibits the NF-kB inflammation pathway and neutrophil adhesion, extravasation, and recruitment by altering neutrophil L-selectin expression and endothelial cell E-selectin distribution. Hypothalamic-hypophyseal-adrenal axis response to stress and its mediators are not presented in this figure. GPCR, G protein-coupled receptors; CNS, Central nervous system; PNS, Peripheral nervous system. Created with BioRender.com.

The Mediterranean fever gene (MEFV), which encodes the pyrin protein, is linked to FMF. Although the mechanisms of pyrin inflammasome activation are not completely understood, one of the most compelling explanations for pyrin regulation is one involving changes in host Rho GTPase activity. PKN1 and PKN2, which are RhoA-dependent serine/threonine protein kinases, directly phosphorylate pyrin at Ser208 and Ser242. Pyrin comes into touch with the chaperone proteins 14-3-3ϵ and 14-3-3 τ as a result of this. This interaction keeps pyrin in an inactive state. When RhoA is inactivated by bacterial toxins or stress mediators, PKN1 and PKN2 activity decreases, resulting in lower amounts of phosphorylated pyrin. This freed pyrin from the inhibitory 14-3-3 proteins, allowing an active pyrin inflammasome to develop faster (28) (**Figure 1**). This will lead to caspase-1 activation and release of IL-1β and IL-18. Col either stimulates RhoA directly or reverses the C3 toxin-induced suppression of RhoA activity (28). It accomplishes this by enhancing the release and activation of guanine-nucleotide-excange factor (GEF)-H1 from depolymerized microtubules (76). Col also controls the innate inflammatory response by inhibiting intracellular signaling pathways such as NF-kB and caspase-1 (77). Col inhibits rolling, adhesion, motility, phagocytic, and some cytokine secretory capabilities of leukocytes, as well as modifies adhesion protein expression (e.g., E-selectin, L-selectin, vascular cell adhesion molecule-1) (78, 79). Furthermore, Col blocks pro-inflammatory cascades by inhibiting the activation of purinergic receptors P2X2 and P2X7 (80) (**Table 1**).

**Table 1:** The effects of colchicine on neuronal and post neuronal levels.

Neuronal effects	Post neuronal anti-inflammatory effect
***Central nervous system*** -It inhibits catecholamine transportation in regions such as LC, PVN where microtubules are dense (53–58) ***Peripheral nervous system*** -It prevents the release of neurotransmitters, particularly noradrenaline, from stimulated sympathetic fibers (59) -It inhibits catecholamine release from the adrenal medulla (60). thereby reducing catecholamine levels (75)	-Modulates adhesion protein expression and functional organization (eg, E-selectin, L-selectin, vascular cell adhesion molecule-1) (78) -Inhibits rolling, adhesion, motility, phagocytic, and certain granule and cytokine secretory functions of leukocytes (79) -Activates RhoA and keeps pyrin in inactive form (28) -Inhibits NF-ĸβ pathway (77) -Suppress the activation of Caspase-1 (8) -Inhibits activation of purinergic receptors P2X2 and P2X7 and blocks pro-inflammatory cascades (80) -Inhibits mast cell histamine release (62)

LC, locus coeruleus; PVN, Paraventricular nuclei.

## Discussion and Future Perspectives

Neuronal regulation of immunity and inflammation is a rapidly growing field, with significant opportunities for new discoveries. It is reasonable that the future will bring new ways of neuromodulator therapy to target diseases currently treated with drugs (81). The abovementioned data suggest that Col can affect the neurosecretory functions of neurons without damaging the neuronal structure. Influencing catecholamine release is one of these effects. In line with this information, the role of Col in preventing stress-induced FMF attacks may be due to its effect on the neural system in addition to anti-inflammatory effects (**Figure 1**).

The fact that Col does not act immediately during an attack may be related to the fact that previously released catecholamines activate inflammation mechanisms until Col penetrates neural tissue. In stressful situations, catecholamines enter the circulation in a very short time. On the other hand, the inhibitory effect of Col on neuronal tissue may be delayed for up to 20-24 hours (82).

Col peaks in plasma 1 hour after oral intake, and a second peak occurs at 6 hours due to enterohepatic circulation (83). The concentration of Col in neutrophils is 60-600 times higher than that in plasma (84), which is because the P-glycoprotein efflux pump system is absent in the neutrophil membrane. The biological effect of a single dose of 1 mg Col appears at 47 hours (85). The late emergence of the biological effect of Col can be suggested as a reason why Col does not show an immediate impact when given during FMF attacks. However, this suggestion seems to be open to question because Col shows its effect quickly in gout attacks. It seems difficult to explain this paradoxical situation with the late emergence of the biological effect of Col in neutrophils. This may be related to the direct effect of Col on the inflammation pathways activated by uric acid. Gout is an IL-1-mediated autoinflammatory disease caused by the activation of the NLRP3 inflammasome and NF-kB pathway (86). Col prevents neutrophil chemotaxis by binding to tubulin and inhibits the initial phase of crystal induced inflammation in gout (87). This feature may provide an advantage to Col to heal gout attacks in a short time. However, a wide variety of neuroendocrine and neuroimmune events develop during stress, and these events affect inflammation pathways through a wide variety of mechanisms (88). Therefore, stress mediators may have multiple roles in activating the inflammasome and other inflammation pathways in FMF rather than a few pathways (89).

It can be speculated that Col may not be equally effective on all pathways; therefore, it cannot end attacks within a short time. Based on the fact that colchicine is effective when given before attacks, not during attacks, Col acts through stress mediators in preventing stress-related FMF attacks.

Our hypothesis regarding the inhibition of the neurosecretory transportation of catecholamines may be an alternative explanation for the prevention of FMF attacks. One of the questions to be asked here is, does this neurosecretory inhibitory effect of Col not affect physiological functions? Interestingly, while this effect of Col is not observed in basal physiological conditions, it is more apparent when the basic secretory activity of the cells is more marked (90).

Given the effect of Col on the CNS and PNS, it is necessary to seek answers to some questions. How do catecholamine levels progress in stress and nonstress periods in FMF patients using or not using Col? Is there a difference in catecholamine levels between Col-resistant FMF patients and Col-responsive FMF patients? Could there be a problem with the neural tissue penetration of Col in Col-resistant patients? How important are MEFV variant differences in the stress response?

In conclusion, in addition to its anti-inflammatory properties at the post neuronal level, Col can prevent the occurrence of stress-related attacks by reducing the transport/release of stress mediators at the neuronal level (**Table 1**). While studies with stress mediators will increase the knowledge on this subject, answering the abovementioned questions will both shed light on the pathogenesis of FMF and lead to new treatment options.

## Data Availability Statement

The raw data supporting the conclusions of this article will be made available by the authors, without undue reservation.

## Author Contributions

CK conceived the idea, formulated the theory, wrote the manuscript. DÜC and GBC contributed original data, discussed the data, and drew the illustrations, and critically revised it. All authors contributed to the article and approved the submitted version.

## Funding

This study has been partially funded by The Turkish Society of Rheumatology.

## Conflict of Interest

The authors declare that the research was conducted in the absence of any commercial or financial relationships that could be construed as a potential conflict of interest.

## Publisher’s Note

All claims expressed in this article are solely those of the authors and do not necessarily represent those of their affiliated organizations, or those of the publisher, the editors and the reviewers. Any product that may be evaluated in this article, or claim that may be made by its manufacturer, is not guaranteed or endorsed by the publisher.
